# Machine learning assisted optimization of blending process of polyphenylene sulfide with elastomer using high speed twin screw extruder

**DOI:** 10.1038/s41598-021-03513-3

**Published:** 2021-12-15

**Authors:** Shingo Takada, Toru Suzuki, Yoshihiro Takebayashi, Takumi Ono, Satoshi Yoda

**Affiliations:** 1 Research Association of High-Throughput Design and Development for Advanced Functional Materials (ADMAT), Higashi 1-1-1, Tsukuba, Ibaraki 305-8565 Japan; 2 Department of Processes Engineering, DIC Corporation, Sakashita 3-35-58, Itabashi, Tokyo 174-8520 Japan; 3 Research Institute for Chemical Process Technology, National Institute of Advanced Industrial Science and Technology (AIST), Higashi 1-1-1, Tsukuba, Ibaraki 305-8565 Japan

**Keywords:** Nanocomposites, Chemical engineering

## Abstract

Random forest regression was applied to optimize the melt-blending process of polyphenylene sulfide (PPS) with poly(ethylene-glycidyl methacrylate-methyl acrylate) (E-GMA-MA) elastomer to improve the Charpy impact strength. A training dataset was constructed using four elastomers with different GMA and MA contents by varying the elastomer content up to 20 wt% and the screw rotation speed of the extruder up to 5000 rpm at a fixed barrel temperature of 300 °C. Besides the controlled parameters, the following measured parameters were incorporated into the descriptors for the regression: motor torque, polymer pressure, and polymer temperatures monitored by infrared-ray thermometers installed at four positions (T1 to T4) as well as the melt viscosity and elastomer particle diameter of the product. The regression without prior knowledge revealed that the polymer temperature T1 just after the first kneading block is an important parameter next to the elastomer content. High impact strength required high elastomer content and T1 below 320 °C. The polymer temperature T1 was much higher than the barrel temperature and increased with the screw speed due to the heat of shear. The overheating caused thermal degradation, leading to a decrease in the melt viscosity and an increase in the particle diameter at high screw speed. We thus reduced the barrel temperature to keep T1 around 310 °C. This increased the impact strength from 58.6 kJ m^−2^ as the maximum in the training dataset to 65.3 and 69.0 kJ m^−2^ at elastomer contents of 20 and 30 wt%, respectively.

## Introduction

Polyphenylene sulfide (PPS) is a super-engineering thermoplastic with excellent thermal stability, chemical resistance, flame retardance, electrical insulation, and mold precision^1–3^. PPS has thus received much attention as an alternative material to metals for automobile and electric parts. However, its brittleness, i.e., poor impact strength, has limited further applications.

Polymer blending is an effective and economical approach to develop new materials with improved properties^4,5^. Blending PPS with elastomer (viscoelastic polymer) can increase the impact strength via energy dissipation by the rubber component^2,3,6–11^. According to recent researches, properties of the PPS/elastomer blend depend not only on the chemical structure and blend ratio of the elastomer, but also on the microscopic morphology of the product^6–11^. This is because the two polymers are immiscible and form a droplet-matrix structure where elastomer particles are dispersed in PPS continuous phase. The more finely the elastomer particles are dispersed with small sizes and short distances, the higher the impact strength is expected to be^12,13^. It is also reported that reactive blending with elastomer having functional groups, such as glycidyl and isocyanate ones, can stabilize the PPS/elastomer interface with good adhesion by the chemical bond formation^6–11^.

Polymer blending is typically done in a melt-mixing process using a twin-screw extruder^14^. The raw polymers are mixed and melted in heated barrels, and then extruded through a cylinder by the rotating screws. The melt-mixing process has many operating parameters, e.g., the polymer composition, feed rate, temperature profile in the cylinder, screw configuration, and its rotation speed. These process parameters affect the properties of the product via the morphology of the polymer blend and the degrees of chemical reactions including thermal degradation. These effects are often interrelated and exhibit nonlinear responses to the target property. It is thus quite time-consuming and costly to optimize the melt-mixing process only by trial and error.

Machine learning, a branch of artificial intelligence (AI), is a promising statistical tool to optimize input parameters in such complicated multivariable systems^15^. Machine learning helps us to find out key parameters, the so-called descriptors, governing the target property and to predict the property as a function of the descriptors. In the field of chemistry, machine learning has been actively employed in ‘materials informatics’ to build design models relating the material properties with the chemical and physical structures from existing datasets or computer simulation results^16–23^. There have been few applications, however, in ‘process informatics’ to clarify how to produce the materials with desired properties^24–27^, especially in the field of polymer industry^28–30^.

Here we used a random forest regression to maximize the impact strength of PPS/elastomer blend. Random forest is a machine learning algorithm based on a decision tree, i.e., a flowchart-like diagram, and can evaluate the importance of each descriptor^31^. Into the descriptors, we incorporated not only the controllable parameters such as the elastomer type, elastomer content, and screw rotation speed, but also the measured ones including the temperature profile, elastomer particle diameter, and melt viscosity of the product. The regression successfully offered us a strategy for systematic optimization of the parameters.

To build a training dataset for the machine learning, we introduced a new high-speed twin-screw extruder equipped with infrared-ray thermometers. This extruder can provide high screw rotation speed up to 6600 rpm, about six times higher than conventional machines. The high shear is effective for the fine dispersion of elastomer particles^32–40^. We should note, however, that large heat of shear may increase the temperature of polymer, leading to a thermal degradation and deteriorated product properties^35–38^. To avoid the overheating, it is necessary to directly monitor the polymer temperature as well as the barrel temperature. Here we installed four infrared thermometers along the cylinder to monitor the polymer temperature profile in situ. Infrared thermometer has the following advantages over thermocouple: quick response, non-intrusive probing without disturbing the polymer flow, and small influence from the surrounding^41–43^. The combination of high-speed extruder and infrared thermometer was found to be a powerful tool in the collection of training data for machine learning. The wide-range control of operating parameter allowed an accurate prediction without extrapolation, while the monitoring of state variables as many as possible enabled a proper selection of the descriptors closely related to the target property.

## Methods

### Raw materials

**Figure 1:**
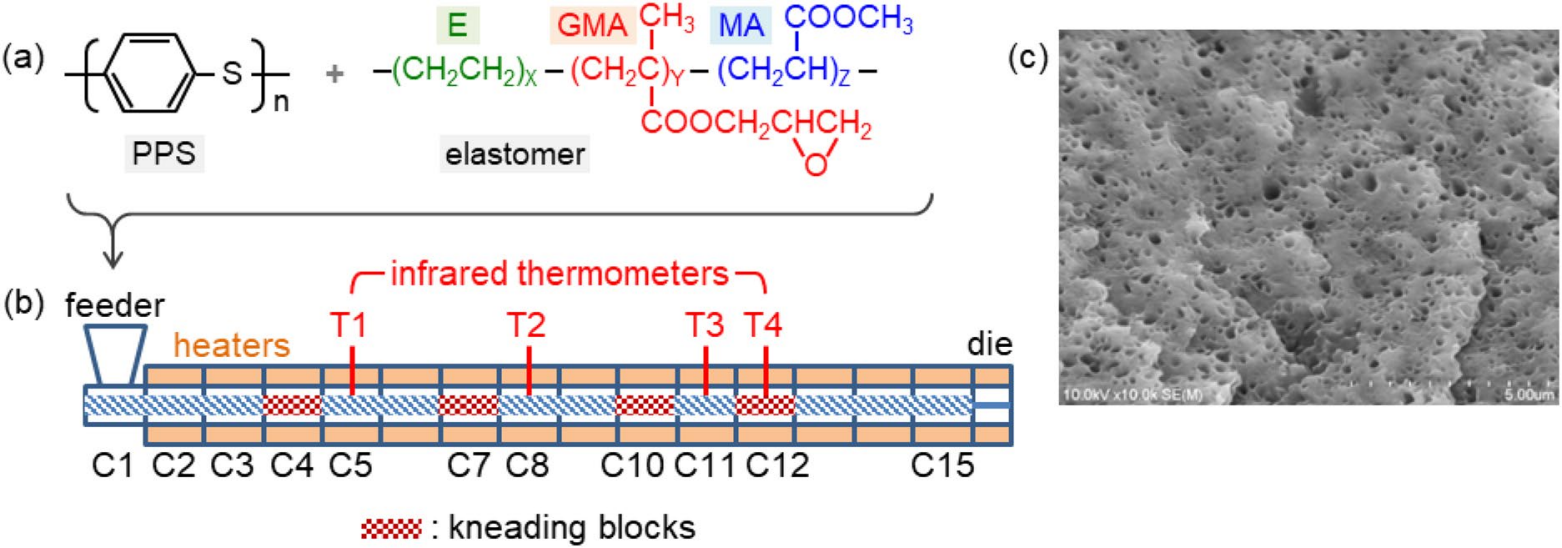
(**a**) Chemical structures of polyphenylene sulfide (PPS) and poly(ethylene-glycidyl methacrylate-methyl acrylate) (E-GMA-MA) elastomer. (**b**) Schematic diagram of the twin-screw extruder. C1 to C15: barrels, T1 to T4: infrared thermometers to monitor the polymer temperatures. (**c**) Typical SEM images of the polymer blend etched by xylene to dissolve elastomer particles.

PPS supplied from DIC Corporation (MA-520) was blended with poly(ethylene-glycidyl methacrylate-methyl acrylate) (E-GMA-MA) elastomer. Chemical structures of the polymers are shown in Fig. 1a. We used four types of elastomers purchased from Sumitomo Chemical (BF-7L, E, 7M, and 2C) with different GMA and MA contents. Specifications of the polymer samples are listed in Table 1.

**Table 1:** Specifications of the polymer samples used.${T}_{\mathrm{g}}$: glass transition temperature, ${T}_{\mathrm{m}}$: melting temperature.

(a) PPS				
Product name	${T}_{\mathrm{g}}$ (°C)	${T}_{\mathrm{m}}$(°C)		
MA-520	95	285		

(b) E-GMA-MA elastomers				
Product name	GMA content (wt%)	MA content (wt%)	${T}_{\mathrm{g}}$ (°C)	${T}_{\mathrm{m}}$ (°C)
BF-7L	3	27	− 33	60
BF-E	12	0	− 26	103
BF-7M	6	27	− 33	52
BF-2C	6	0	− 26	105

### Melt blending with twin-screw extruder

The PPS and elastomer were mixed at various ratios from 98/2 to 80/20 wt%. The mixture was fed into a high-speed twin-screw extruder (Technovel, MFU-15TW-90MG-NH(-6600)-SST) at a constant feed rate of 50 g min^−1^. The extruder had a screw length $L$ of 1350 mm and a screw diameter $D$ of 15 mm, yielding the $L/D$ ratio of 90. The screw rotation speed was varied from 150 to 5000 rpm. The extruder consisted of 15 barrels, as illustrated in Fig. 1b, numbered from C1 on the feeder side to C15 on the die side. Four kneading blocks were located at the barrels C4, C7, C10, and C12. Each kneading block was composed of four pieces of five-disc element with a staggering angle of 45°. The barrels C2 to C15 were covered by electric heaters. Temperatures of all the barrels were controlled to 300 °C during the preparation of training dataset. Polymer temperature was measured at four positions with infrared thermometers (Futaba Corporation, EPSSZL) T1 to T4 inserted into the barrels C5, C8, C11, and C12, respectively. In addition to the polymer temperature, the polymer pressure and the motor torque current were monitored under each experimental condition and were used as the descriptors for machine learning. The polymer pressure was measured at the die head. The die was a dual strand die with hole diameter of 2.5 mm. The product was cooled by air and was pelletized.

### Characterizations of the polymer blend

Charpy notched impact strength of the polymer blend was measured at 23 °C with a digital impact tester (Toyo Seiki) according to ISO 179-1. The test piece (80 mm length, 10 mm width, and 4.0 mm thickness) was prepared with an injection molding machine (Sodic, LP20EH3) at a cylinder temperature of 300 °C and a mold temperature of 130 °C. The test piece was notched by 2 mm according to ISO 2818.

Morphology of the polymer blend was observed with a scanning electron microscope (SEM) (Hitachi High-Technologies, S-4800). Prior to the SEM observation, a test piece molded according to ISO 3167-A was cooled in liquid nitrogen and was cryo-fractured by bending. The fracture surface was etched by xylene at 50 °C for 1.0 h in an ultrasonic bath to selectively dissolve the elastomer particles dispersed in the PPS matrix. The sample was then dried at 130 °C for 2.0 h and was coated with Pt–Pd using an ion sputter coater (Hitachi High-Technologies, E-1010) for the SEM observation. Typical SEM image is shown in Fig. 1c. Area of each void in the SEM image was measured using ImageJ software^44^ (version 1.53) to calculate the corresponding elastomer particle diameter. The elastomer particle diameter was averaged over more than 300 voids to determine the mean value.

Melt viscosity of the polymer blend was measured with a constant-force-type capillary rheometer (Shimadzu, CFT-500D). The sample was preheated at 300 °C for 6.0 min and then was extruded through a capillary (1 mm diameter and 10 mm length) at a pressure of 4.9 MPa. It is to be noted that the melt viscosity of polymer is a function of the shear rate, and thus should be compared at a constant shear rate for quantitative discussion. The melt viscosity change measured here at a constant pressure is an apparent one. We can estimate, however, the real viscosity change from the apparent one and can expect that the real viscosity change is qualitatively similar to the apparent one enough to discuss the increase or decrease in the molecular weight. The detail of the estimation is provided in Supporting Information.

### Machine learning

Machine learning was performed using python software^45^ (version 3.7) with scikit-learn library^46^ (version 0.23.1). Random forest regression of the Charpy impact strength was carried out to evaluate the importance of the parameters listed in Table 2. No prior knowledge was employed for the regression. In addition, nonlinear support vector machine was used to regress the polymer temperature T1 as a function of the elastomer content and the screw rotation speed. The regression was done after standardization of the two descriptors using a radial basis function kernel with the following hyperparameters: the penalty parameter $C$ = 1000 and the kernel coefficient $\gamma$ = 0.05.

**Table 2:** List of the parameters and their ranges of variation.

Parameters	Range
**Controlled parameters**	
Elastomer content in polymer blend (wt%)	2, 5, 10, 15, 20 (= *a*)
GMA content in elastomer (wt%)	3, 6, 12 (= *b*)
MA content in elastomer (wt%)	0, 27 (= *c*)
GMA content in polymer blend (wt%)	*a* × *b*/100
MA content in polymer blend (wt%)	*a* × *c*/100
Screw rotation speed (rpm)	150, 300, 600, 1000, 1500, 2000, 2500, 3000, 3500, 4000, 4500, 5000
**Measured parameters—process**	
Motor torque current (A)	67.0–98.7
Polymer pressure (MPa)	0.1–5.7
Polymer temperature T1 (°C)	305–417
Polymer temperature T2 (°C)	307–393
Polymer temperature T3 (°C)	303–369
Polymer temperature T4 (°C)	305–389
**Measured parameters—product**	
Elastomer particle diameter (nm)	52–544
Melt viscosity (Pa s)	50–9880
Charpy impact strength (kJ m^−2^)	1.1–58.6

## Results and discussions

### Charpy impact strength

**Figure 2:**
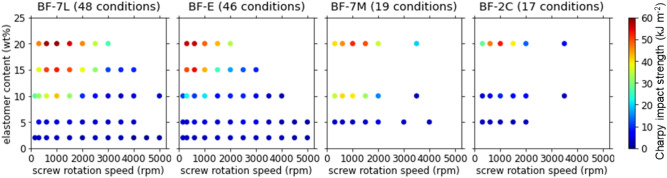
Charpy impact strength of the polymer blend as a function of the elastomer type, elastomer content, and screw rotation speed.

Figure 2 describes how the impact strength of the polymer blend varied with the elastomer type, elastomer content, and screw rotation speed. Among the 130 experimental conditions, the Charpy impact strength ranged widely from 1.1 to 58.6 kJ m^−2^. In most conditions, the impact strength was lower than 20 kJ m^−2^. It should be noted, however, that the ‘failed’ results also played an essential role in the following machine learning. Changes in the other measured parameters are summarized in Supplementary Information S1.

**Figure 3:**
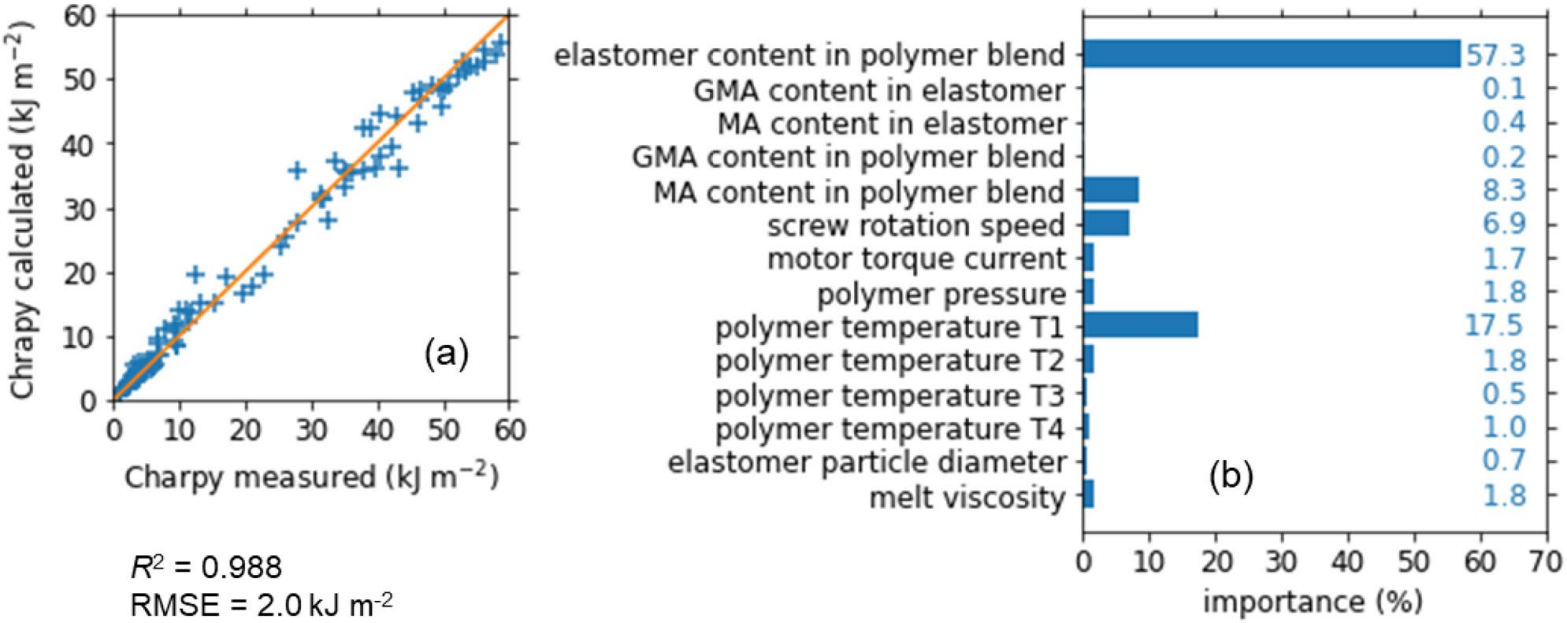
Random forest regression of the Charpy impact strength. (**a**) Calculated values plotted against the measured one. (**b**) Importance of the parameters in the regression.

Figure 3 shows the results of the random forest regression of the Charpy impact strength. Correlation coefficient of the regression ${R}^{2}$ was as high as 0.988 and the root-mean-squared error (RMSE) was 2.0 kJ m^−2^. Importance of the parameters in the regression is shown in Fig. 3b. The elastomer content had the highest importance over 50%. The next most important parameter was the polymer temperature T1 just after the first kneading block. The importance of T1 was as high as 17%. This result means that polymer temperature in the cylinder can be a key parameter in melt-blending process, although it has been rarely measured in most extruders reported so far. For the rest parameters, the importance was lower than 10%. Thus, the impact strength is determined mainly by the two key parameters: the elastomer content and the polymer temperature T1, as is confirmed later. In contrast, the elastomer type, i.e., the GMA and MA contents in elastomer, was much less important than the two key parameters. In fact, the impact strength exhibited qualitatively similar behavior among the four elastomers tested. In the latter sections, therefore, our discussion is focused on the result for elastomer BF-7L as a representative one.

**Figure 4:**
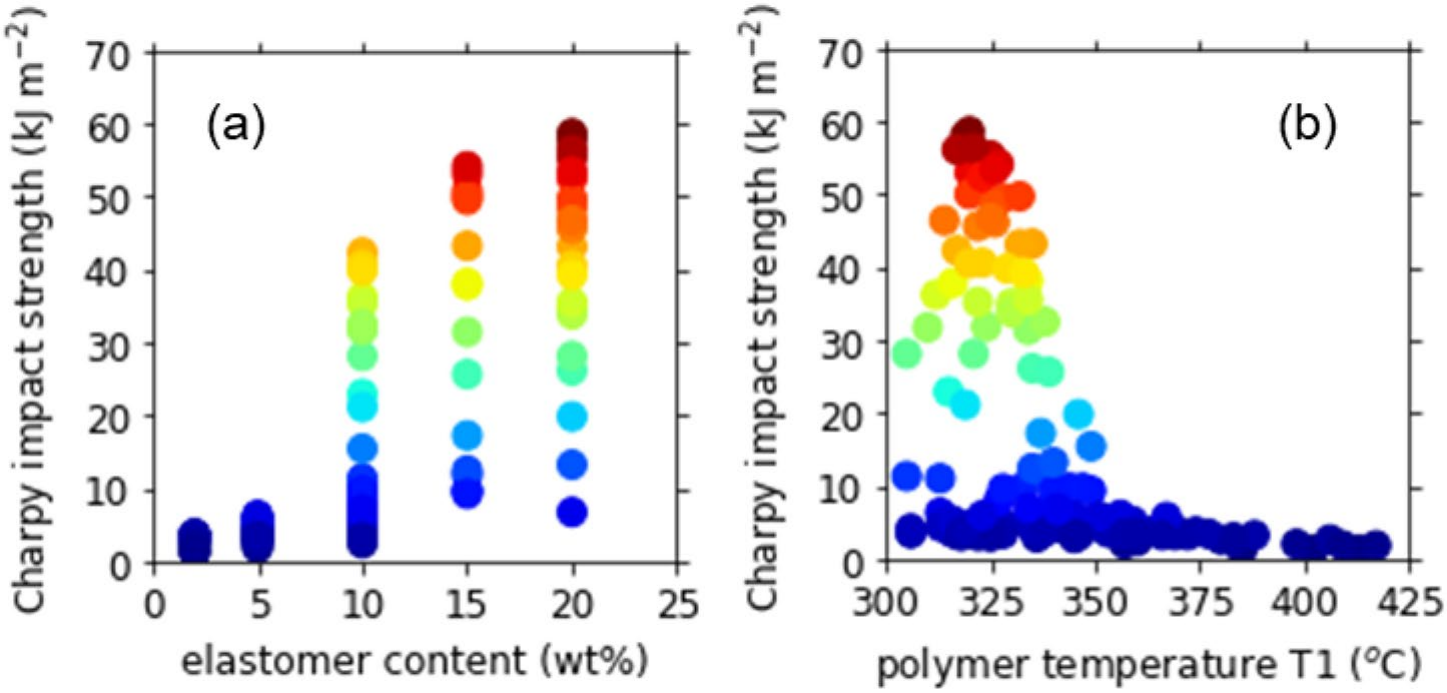
Charpy impact strength plotted against (**a**) the elastomer content and (**b**) the polymer temperature T1.

In Fig. 4, the Charpy impact strength is plotted against the two key parameters. Figure 4a indicates that high elastomer content was a necessary condition to obtain high impact strength, although it was not a sufficient condition. The maximum impact strength increased monotonically with the elastomer content. We can thus expect further high impact strength at elastomer content above 20 wt%. Against the polymer temperature T1, as shown in Fig. 4b, the impact strength had a sharp maximum around 320 °C. When T1 was within 320 ± 10 °C and the elastomer content was higher than 15 wt%, the impact strength was never lower than 20 kJ m^−2^. With increasing T1 above 330 °C, however, the impact strength decreased markedly to less than 5 kJ m^−2^. The temperature dependence is interpreted in terms of the polymer degradation in the next section. The decrease in the impact strength was observed also at low T1. This is due to the low screw rotation speed. As described in the next section, the polymer temperature T1 was strongly correlated with the screw rotation speed, when the barrel temperature was fixed at 300 °C. The lower the screw rotation speed, the lower the polymer temperature T1. At the low screw rotation speed, the shearing was insufficient for fine dispersion of elastomer particles, leading to the low impact strength at low T1.

### Polymer temperature

**Figure 5:**
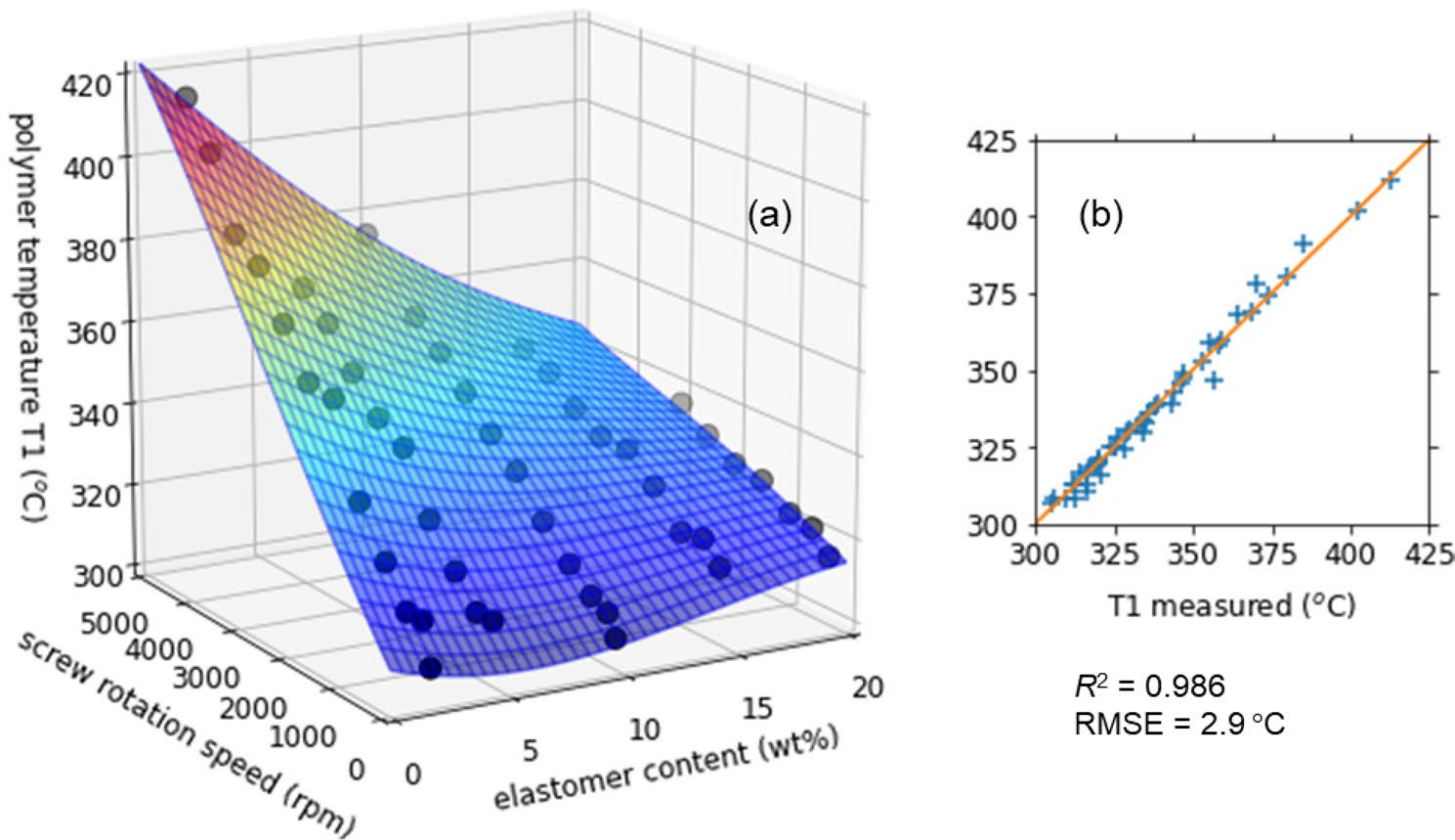
(**a**) Polymer temperature T1 as a function of the elastomer content and the screw rotation speed in the melt-blending process of PPS/BF-7L at a fixed barrel temperature of 300 °C. The curved surface was obtained by a nonlinear support vector regression of the experimental data. (**b**) T1 calculated from the regression plotted against the measured one.

The polymer temperature T1 is plotted in Fig. 5 as a function of the elastomer content and the screw rotation speed. T1 exhibited a monotonic increase with increasing screw rotation speed due to the heat of high shear^35–38^. The temperature increase was more evident at lower elastomer content. The polymer temperature T1 reached 417 °C at the maximum, far above the barrel temperature (300 °C). Interestingly, the maximum temperature of T1 was higher than those of T2 to T4 (369 to 393 °C). The result suggests that the overheating is caused not only by the shear heat but also by the exothermic reactions, such as the bond formation at the glycidyl group of GMA and the oxidation by air^3^ occurring more intensely on the feeder side due to the higher concentration of oxygen.

Since the experimental data of T1 is discrete, we interpolated it by a nonlinear support vector regression, as described by the curved surface in Fig. 5. The regressed result can serve as a ‘soft sensor’ to predict the polymer temperature in the cylinder from the elastomer content, screw rotation speed, and barrel temperature^41^. Correlation coefficient ${R}^{2}$ of the regression was as high as 0.986 and the RMSE was smaller than 3 °C. In the following optimization, the regression eased us to estimate the barrel temperature to adjust the polymer temperature T1 around a desired value.

### Melt viscosity and particle diameter

**Figure 6:**
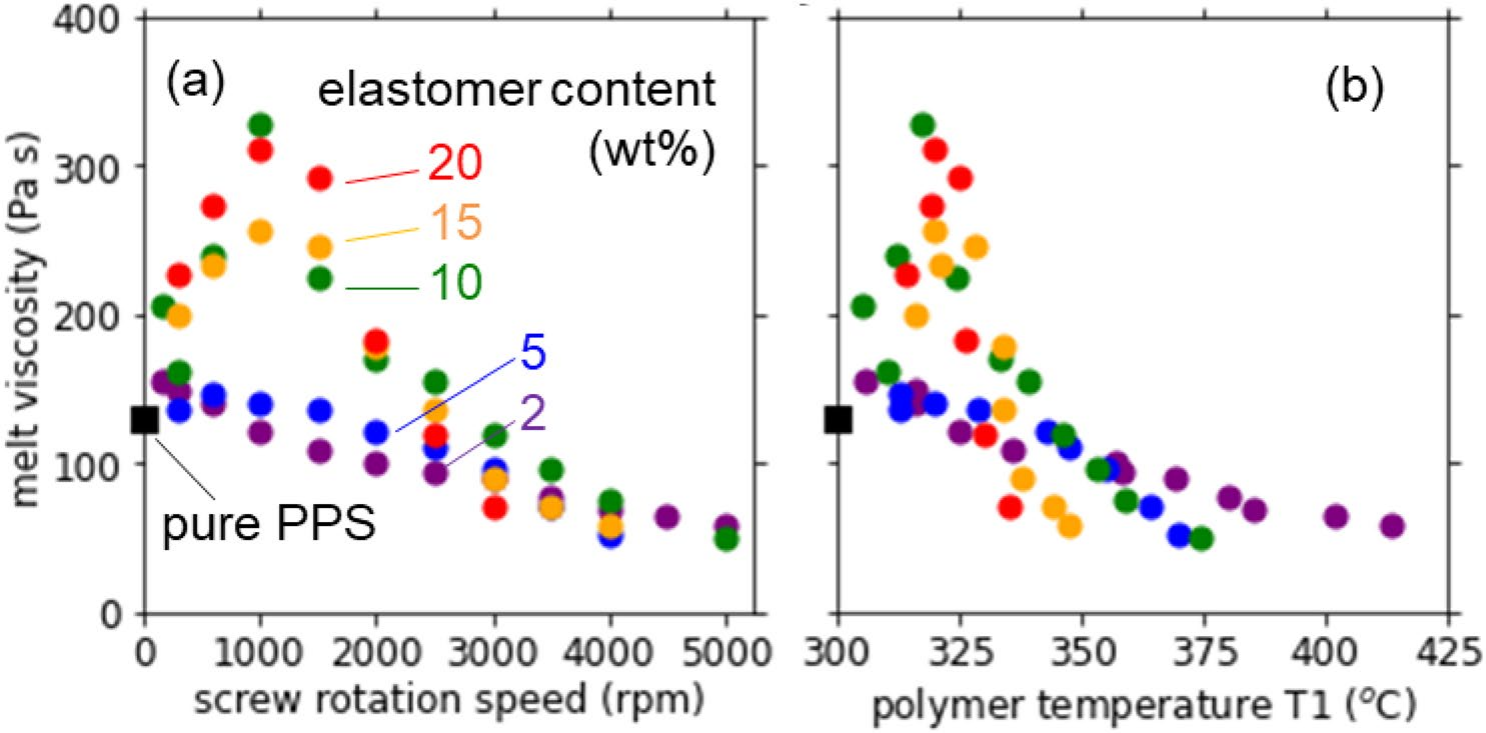
Melt viscosity of the PPS/BF-7L blend as a function of (**a**) the screw rotation speed and (**b**) the polymer temperature T1 at various elastomer contents.

To understand the influence of overheating on the product, the melt viscosity is plotted in Fig. 6 against the screw rotation speed and the polymer temperature T1. With increasing screw rotation speed or T1, the melt viscosity increased markedly from the value of pure PPS (130 Pa s) to more than 250 Pa s at 1000 rpm or 320 °C when the elastomer content was higher than 10 wt%. The increase in the melt viscosity shows an increase in the molecular weight of polymer blend due to the bond formation between PPS and glycidyl group of the elastomer^9–11^. At higher screw rotation speed or T1, however, the melt viscosity turned to decrease down to 60 Pa s. The evident decrease in the melt viscosity indicates a degradation of polymer probably due to a thermal decomposition by the overheating as well as a mechanochemical one by the high shear. The degradation is a main reason why the impact strength decreased at high screw rotation speed or T1. In our subsequent work, the polymer degradation is investigated in more detail by near infrared and Raman spectroscopies.

**Figure 7:**
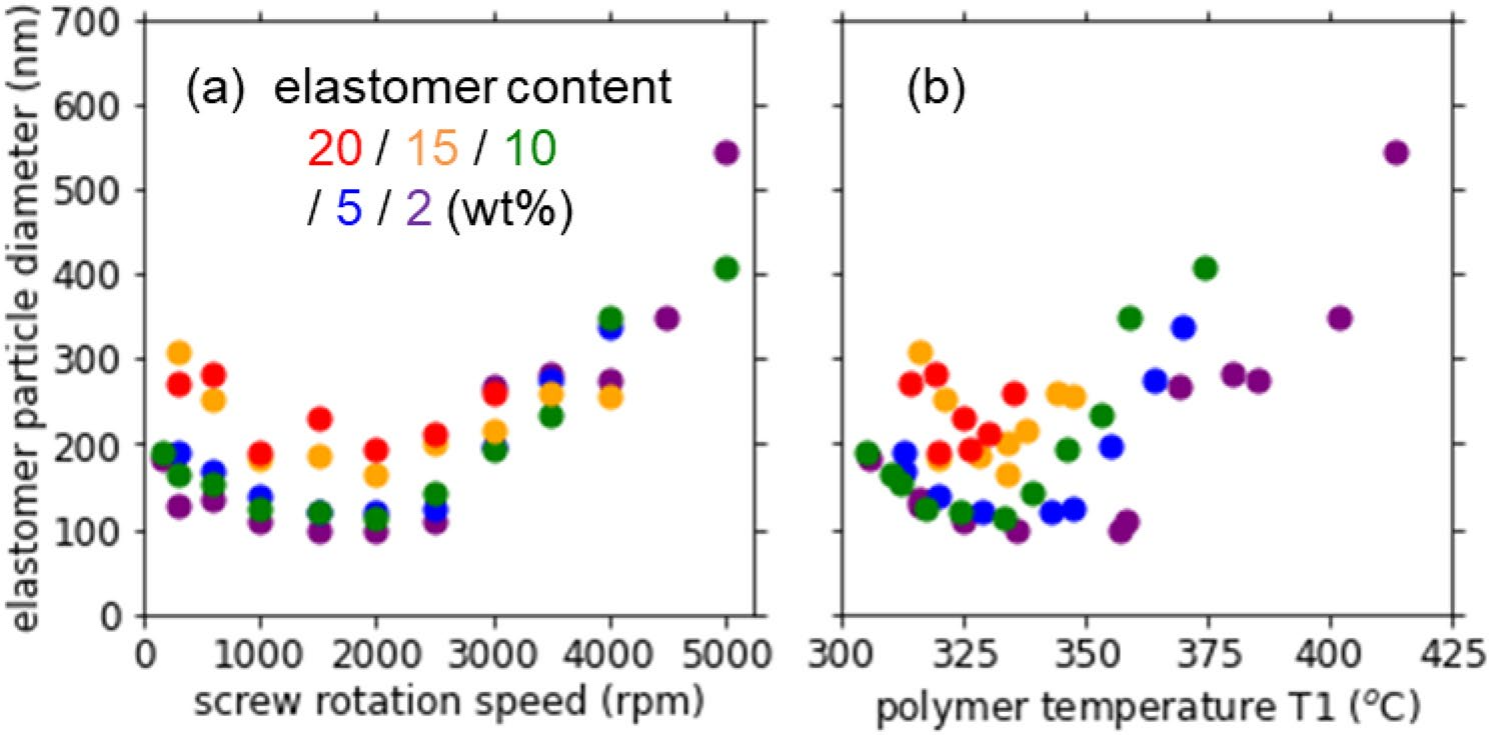
Elastomer particle diameter of the PPS/BF-7L blend as a function of (**a**) the screw rotation speed and (**b**) the polymer temperature T1 at various elastomer contents.

In Fig. 7, we further plot the elastomer particle diameter against the screw rotation speed and T1. The elastomer diameter decreased initially with increasing screw rotation speed up to 2000 rpm at all the elastomer contents due to the enhanced shearing. The fine dispersion of elastomer particle would improve the impact strength. At higher screw rotation speed, however, the particle diameter turned to increase probably because of the polymer degradation. The large particle diameter is another reason for the decreased impact strength at high screw rotation speed or T1.

### Optimization

**Figure 8:**
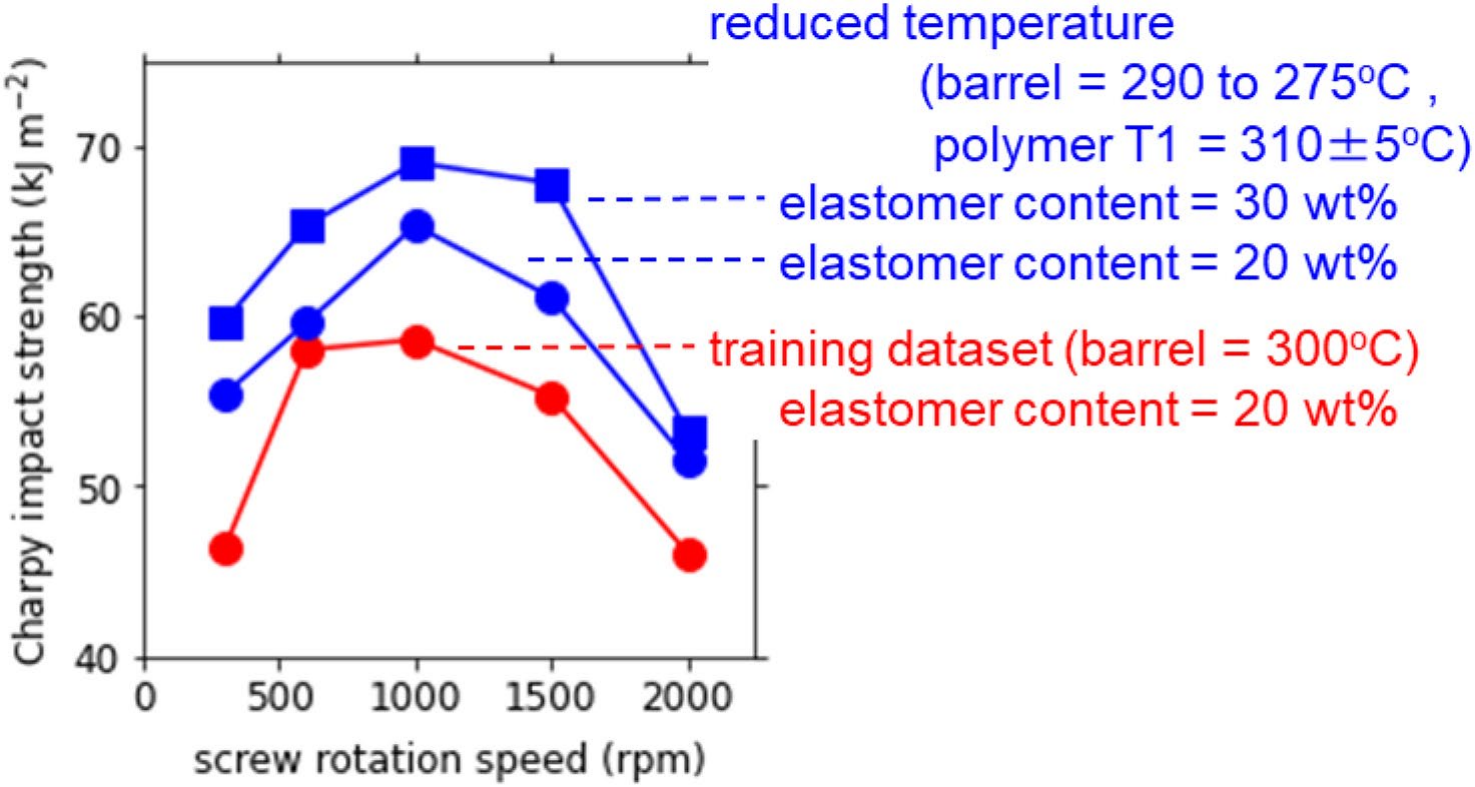
Charpy impact strength of the PPS/BF-7L blend as a function of the screw rotation speed at the elastomer contents of 20 and 30 wt% when the barrel temperature was reduced to 290 to 275 °C to keep the polymer temperature T1 around 310 ± 5 °C. The training dataset where the barrel temperature was fixed at 300 °C is also shown for comparison.

To avoid the polymer degradation by the overheating, we reduced the barrel temperature by 10 to 25 °C so that the polymer temperature T1 was within 310 ± 5 °C at each screw rotation speed from 300 to 2000 rpm, as shown in Supporting Information. The results are compared in Fig. 8 with those of the training dataset where the barrel temperature was fixed at 300 °C. By the temperature reduction at the elastomer content of 20 wt%, the impact strength increased from 58.6 kJ m^−2^ to 65.3 kJ m^−2^ at the maximum. The impact strength had a maximum at 1000 rpm and decreased at higher screw rotation speed, suggesting that the polymer degradation occurs not only thermally by the shear heat but also mechanochemically by the shearing itself. We further increased the elastomer content from 20 to 30 wt% at the reduced temperature condition and obtained higher impact strength of 69.0 kJ m^−2^. The result supports our strategy proposed by the random forest analysis.

## Conclusion

We applied a random forest regression for the optimization of melt-blending process of PPS and elastomer with a high-speed twin-screw extruder. The regression gave us a strategy that high impact strength is obtained at high elastomer content and low polymer temperature below 320 °C. The latter condition was found to be important to avoid the polymer degradation by the overheating due to heat of shear. By reducing the barrel temperature to adjust the polymer temperature T1 around 310 °C, the Charpy impact strength was improved up to 69.0 kJ m^−2^. The present approach is quite general and needs no prior knowledge. This method will be thus applicable for a wide variety of engineering processes to find key parameters, tune multivariable recipes, shorten the development time, and gain deep insight into hidden relationship.

## Supplementary Information

Supplementary Information.
