# Supplementary material for: Machine learning assisted optimization of blending process of polyphenylene sulfide with elastomer using high speed twin screw extruder
Source: Sci Rep. 2021 Dec 15;11:24079. doi: 10.1038/s41598-021-03513-3 (PMC8674312; doi:10.1038/s41598-021-03513-3)
Supplement: Supplementary file 1 — Supplementary Information. [file 41598_2021_3513_MOESM1_ESM.pdf]

*Supplementary Information*

# Machine Learning Assisted Optimization of Blending Process of Polyphenylene Sulfide with Elastomer Using High Speed Twin Screw Extruder

Shingo Takada[1, 2], Toru Suzuki[2], Yoshihiro Takebayashi[3]*, Takumi Ono[3], and Satoshi Yoda[3]

[1] Research Association of High-Throughput Design and Development for Advanced Functional Materials (ADMAT), Higashi 1-1-1, Tsukuba, Ibaraki 305-8565, Japan

[2] Department of Processes Engineering, DIC Corporation, Sakashita 3-35-58, Itabashi, Tokyo 174-8520, Japan

[3] Research Institute for Chemical Process Technology, National Institute of Advanced Industrial Science and Technology (AIST), Higashi 1-1-1, Tsukuba, Ibaraki 305-8565, Japan

* Corresponding author. Phone: +81-29-861-9272. E-mail: y-takebayashi@aist.go.jp.

**S1. Changes in the measured parameters**

**S2. Melt viscosity as a function of the shear rate**

**S3. Change in the polymer temperature T1 upon the optimization**

## S1. Changes in the measured parameters

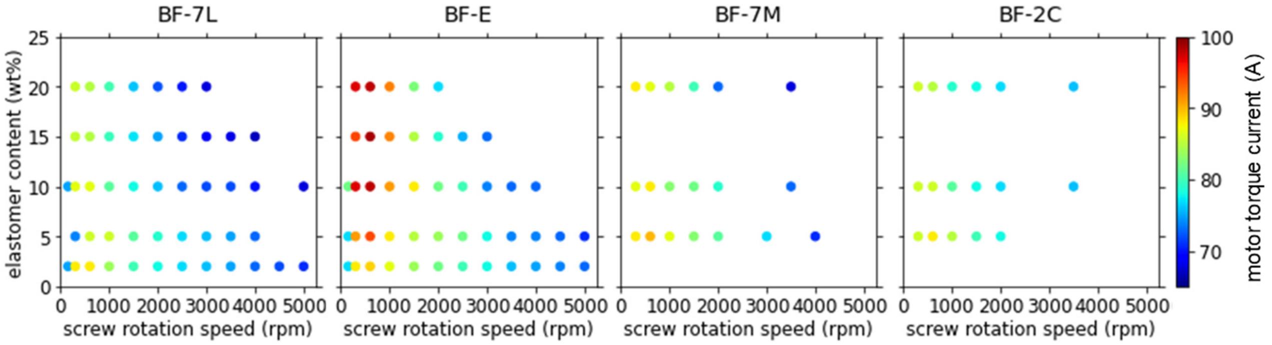


**Figure S1.** Motor torque current as a function of the elastomer type, elastomer content, and screw rotation speed.

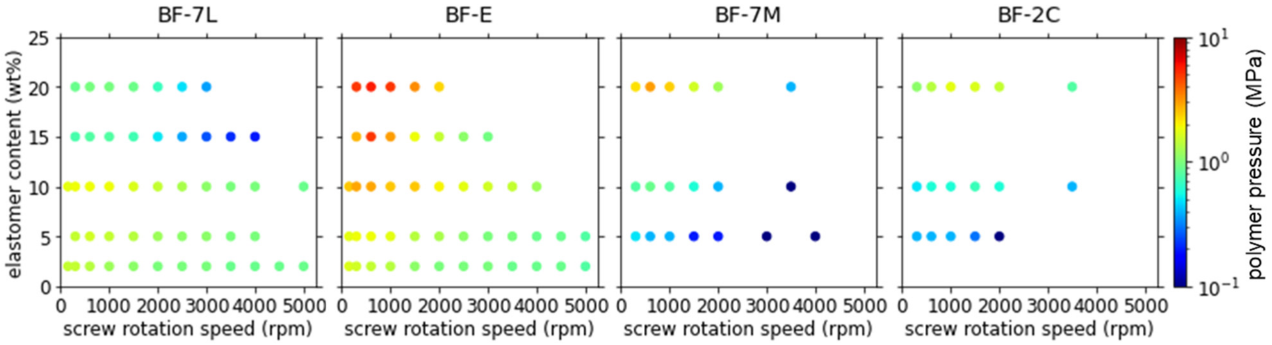


**Figure S2.** Polymer pressure as a function of the elastomer type, elastomer content, and screw rotation speed.

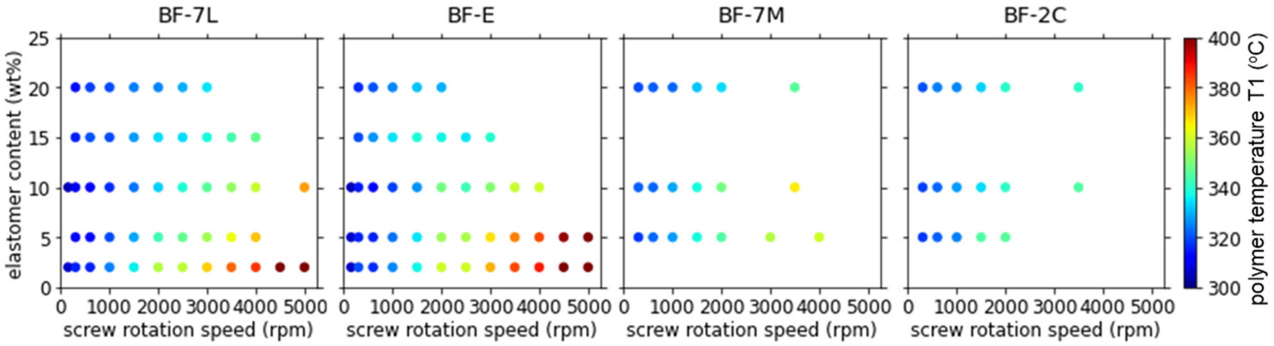


**Figure S3.** Polymer temperature T1 as a function of the elastomer type, elastomer content, and screw rotation speed.

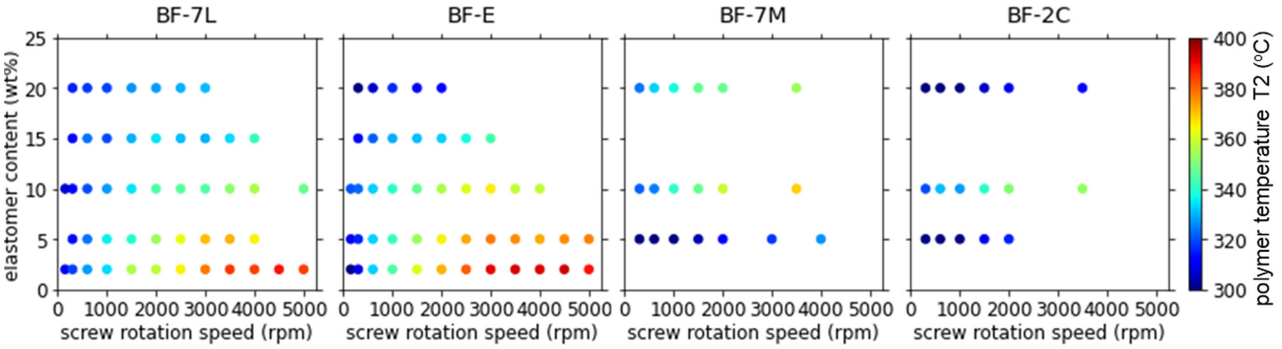


**Figure S4.** Polymer temperature T2 as a function of the elastomer type, elastomer content, and screw rotation speed.

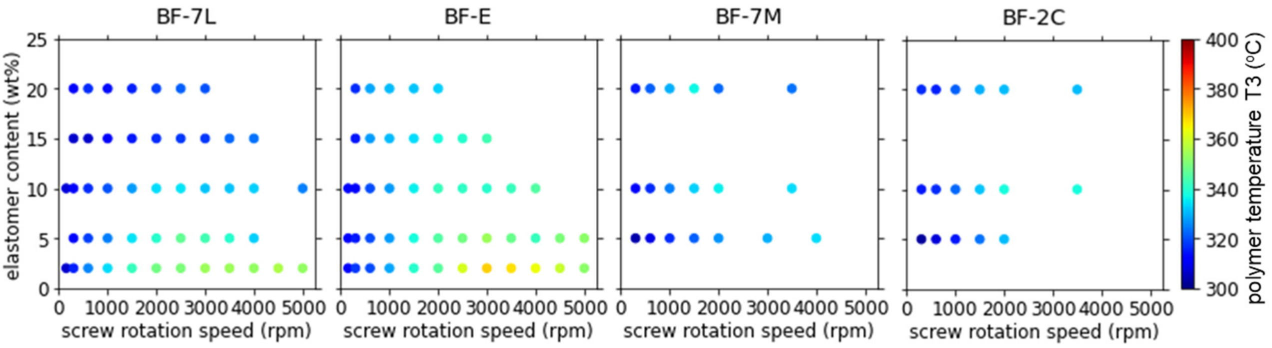


**Figure S5.** Polymer temperature T3 as a function of the elastomer type, elastomer content, and screw rotation speed.

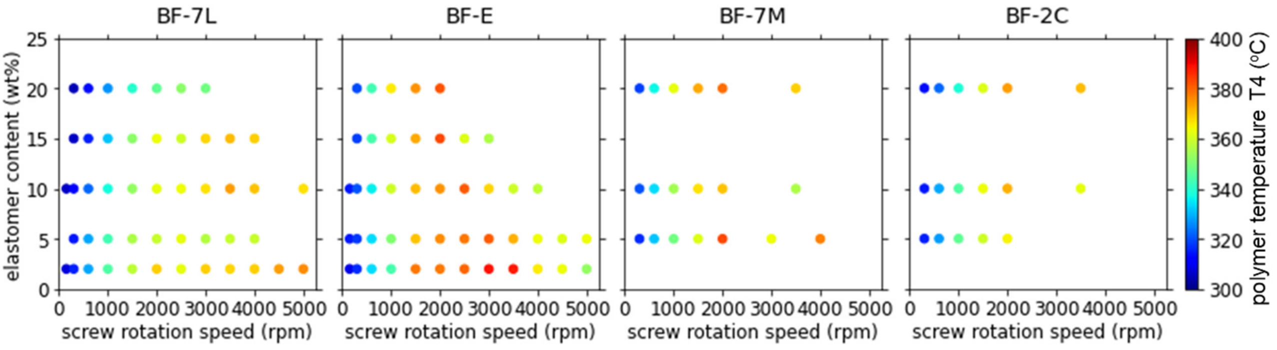


**Figure S6.** Polymer temperature T4 as a function of the elastomer type, elastomer content, and screw rotation speed.

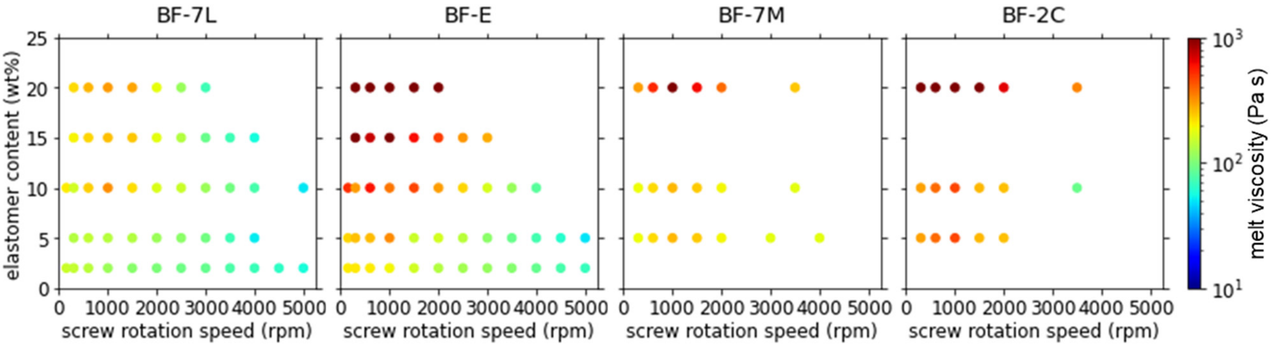


**Figure S7.** Melt viscosity of the polymer blend as a function of the elastomer type, elastomer content, and screw rotation speed.

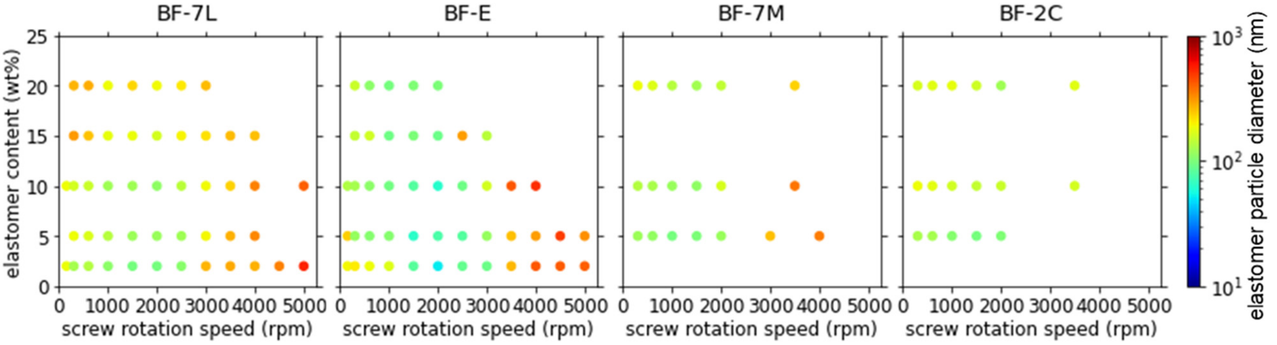


**Figure S8.** Elastomer particle diameter of the polymer blend as a function of the elastomer type, elastomer content, and screw rotation speed.

**S2. Melt viscosity as a function of the shear rate**

Melt viscosity of polymer is a function of the shear rate. Figure S9 shows the melt viscosity of PPS measured with a plunger-type capillary rheometer (Toyo Seiki, Capillograph 1D; inner diameter and length of the capillary were 1 and 40 mm, respectively) at various shear rates and 300 ºC. The melt viscosity of PPS was a decreasing function of the shear rate, as is generally observed for other polymers. The change in the melt viscosity $\eta$ (Pa s) with the shear rate $\gamma$ ($s^{-1}$) can be expressed by the power function:

$$\eta \propto \gamma^{-\alpha}, \qquad \text{(S1)}$$

where the exponent $\alpha$ is a constant and was 0.177 for PPS.

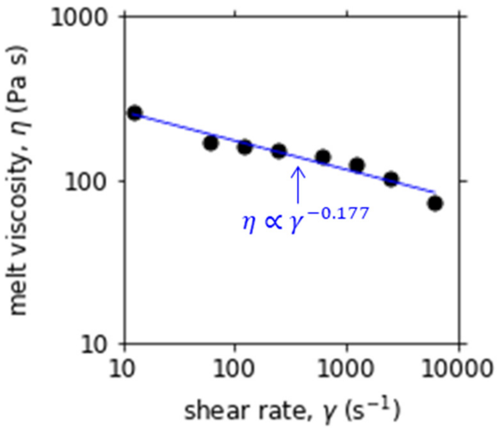


**Figure S9.** Melt viscosity of PPS at 300 ºC as a function of the shear rate.

For quantitative discussion, therefore, the melt viscosity should be compared at a constant shear rate. In the present study, in contrast, the melt viscosity was measured at a constant pressure of 4.9 MPa with a constant-force-type capillary rheometer (Shimadzu, CFT-500D). Thus, the viscosity change presented in this paper is an apparent one. We can estimate, however, the real viscosity change from the apparent one and can expect that the real viscosity change is qualitatively similar to the apparent one enough to discuss the increase and decrease in the molecular weight, as explained in the following.

In the measurement with a capillary rheometer, the melt viscosity $\eta$ (Pa s) is obtained by

$$\eta = \frac{PD}{4L\gamma} = \frac{\pi PD^4}{128LQ}, \tag{S2}$$

where $P$ (Pa) is the pressure, $D$ (m) and $L$ (m) are the inner diameter and length of the capillary, $\gamma$ (s$^{-1}$) is the shear rate, and $Q$ (m$^3$ s$^{-1}$) is the volume flow rate. Equation (S2) shows that the melt viscosity $\eta$ is inversely proportional to the shear rate $\gamma$, when the pressure $P$ is fixed.

Let us suppose that the melt viscosity measured at a constant shear rate $\gamma_0$ varies from $\eta_0$ to $\eta'_0$, while the apparent melt viscosity measured at a constant pressure varies from $\eta_0$ to $\eta'_{\mathrm{app}}$, as illustrated in Figure S10. Assuming that the shear rate dependence of the melt viscosity $\eta \propto \gamma^{-\alpha}$ is negligibly affected by the viscosity change (otherwise the comparison at a constant shear rate is also meaningless), the apparent shear rate $\eta'_{\mathrm{app}}$ is related to the corresponding shear rate $\gamma'$ by

$$\eta'_{\mathrm{app}} = \eta'_0(\gamma'/\gamma_0)^{-\alpha}. \tag{S3}$$

Since the melt viscosity is inversely proportional to the shear rate at a constant pressure,

$$\eta'_{\mathrm{app}}\gamma' = \eta_0\gamma_0. \tag{S4}$$

Combining these equations, we can estimate the real change in the melt viscosity $\eta'_0/\eta_0$ from the apparent one $\eta'_{\mathrm{app}}/\eta_0$ by

$$\eta'_0/\eta_0 = \left(\eta'_{\mathrm{app}}/\eta_0\right)^{1-\alpha} \tag{S5}$$

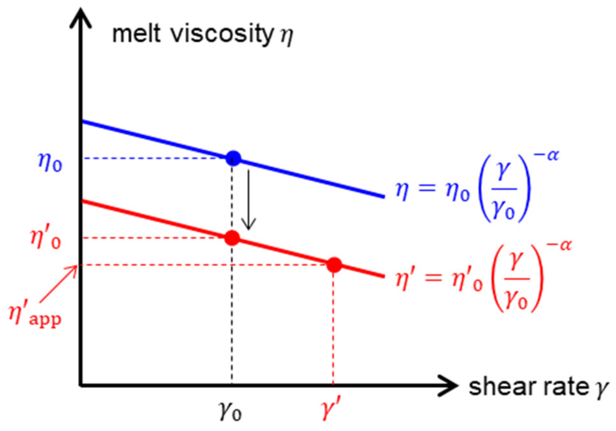


**Figure S10.** Relationship between the apparent melt viscosity change and the real one.

In Figure S11, the real melt viscosity calculated with Equation (S5) using $\alpha$ = 0.177 and $\eta_0$ = 130.2 (Pa s) is compared with the apparent one. The real melt viscosity shows qualitatively similar behavior to the apparent one, although the magnitude of the change is overestimated in the apparent melt viscosity.

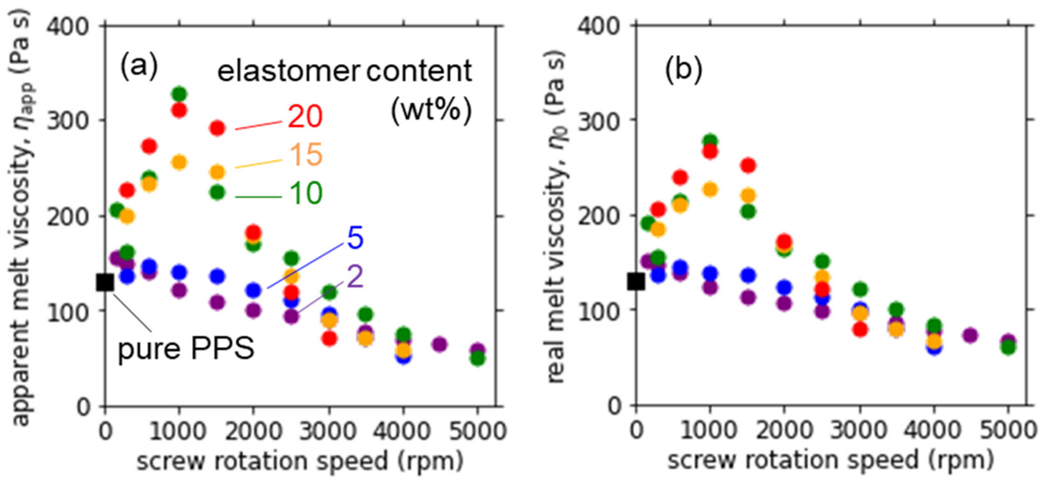


**Figure S11.** (a) Apparent melt viscosity of the PPS/BF-7L blend as a function of the screw rotation speed (the same with Figure 6a) and (b) the real one calculated with Equation (S5).

## S3. Change in the polymer temperature T1 upon the optimization

In Section 3.4, we reduced the barrel temperature to avoid the degradation of polymer so that the polymer temperature T1 was within 310 ± 5 °C at each screw rotation speed. The change in the barrel temperature and the resultant change in the polymer temperature T1 are plotted in Figure S12 as functions of the screw rotation speed.

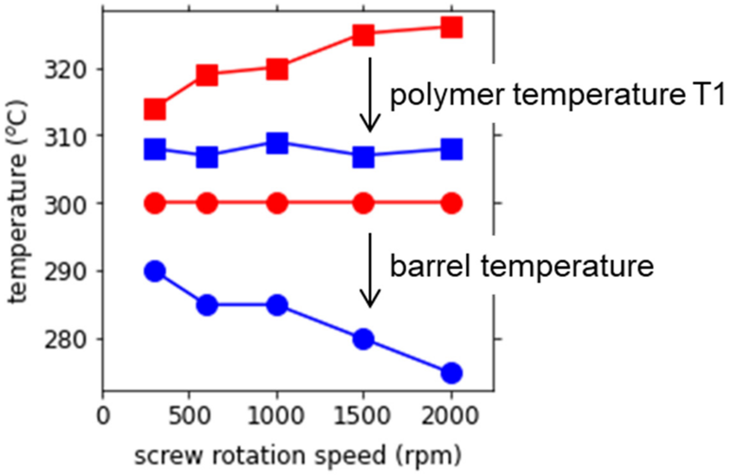


**Figure S12.** Change in the polymer temperature T1 (■: red → blue) upon the reduction in the barrel temperature (●: red → blue) at various screw rotation speeds for the PPS/BF-7L blend at the elastomer content of 20 wt%.
